# Social Perception in Schizophrenia: Evidence of Reduced Prejudiced Attitudes Among People With a Diagnosis of Schizophrenia

**DOI:** 10.3389/fpsyg.2021.644488

**Published:** 2021-04-27

**Authors:** Luigi Castelli, Luciana Carraro, Alessia Valmori, Chiara Uliana, Massimiliano Paparella

**Affiliations:** ^1^Department of Social and Developmental Psychology, University of Padua, Padua, Italy; ^2^Independent Researcher, Pordenone, Italy

**Keywords:** intra-minority perception, schizophrenia, prejudice, social cognition, lay beliefs

## Abstract

Only recently research in social psychology has started to systematically investigate intergroup attitudes among members of stigmatized minority groups. In particular, the study of the way people with mental health problems perceive the social groups around them is so far very scarce. In this work, we focused on people with schizophrenia, analyzing their attitudes toward another stigmatized group, namely Black individuals. In Study 1, the attitudes toward White and Black people were assessed in a sample of respondents with a diagnosis of schizophrenia and in a sample of non-clinical individuals. Results showed the presence of less negative attitudes toward the minority outgroup (i.e., Black people) among the clinical sample. In Study 2, we aimed at investigating what members belonging to the majority group (i.e., White non-clinical people) believe about the attitudes toward Black people held by individuals with a diagnosis of schizophrenia. In general, results suggested a general awareness in lay persons that people with a diagnosis of schizophrenia, as compared to people with no history of mental disorders, hold reduced negative attitudes toward Black individuals. Overall, these results may help to enrich our knowledge about social cognition among members of stigmatized groups in general and, more specifically, among individuals with a diagnosis of schizophrenia.

## Introduction

Research in social psychology has widely investigated both the contents and processes related to the attitudes that majority groups hold toward stigmatized minority groups and, more recently, the attitudes of minority group members toward their own group and toward majority groups (see Brown, 2010). However, the analysis of the attitudes that members of minority groups display toward other minority groups is still largely neglected (Craig and Richeson, 2016). In the present work, we first focused on how members of a strongly stigmatized group, namely individuals with schizophrenia, evaluate the members of another negatively perceived group in their social context, namely Black people. Next, we examined lay people’s expectations about the attitudes hold by individuals with schizophrenia toward Black people. This may inform theories about group perception and, in addition, it may enable to enrich the knowledge about social perception processes in schizophrenia.

### Intra-Minority Attitudes

At a very general level, two opposite predictions can be put forward in relation to the impact that being a member of a stigmatized group might have on the perception of other stigmatized groups. On the one hand, experiences of discrimination may motivate intergroup bias (see Hogg and Abrams, 1990; Branscombe et al., 1999). By creating positive distinctiveness for their group (i.e., perceiving the outgroup as more negative than one’s own group), people may bolster a threatened personal and group esteem. There is significant experimental evidence in support of this view. For instance, Craig and Richeson (2014) have shown that reminding Black and Latino participants about the discriminatory behaviors their respective groups are often targets of, may lead to more negative attitudes toward other minority groups, such as homosexual people. Similarly, making gender discrimination salient leads White women to display stronger racial bias against Black and Latino individuals (Craig and Richeson, 2014).

On the other hand, other studies have shown that when the two minority groups are discriminated in relation to the same underlying dimension (e.g., they are both discriminated for their ethnicity), intra-minority perceptions tend to be more positive. For instance, Craig and Richeson (2012) found that Asian Americans and Latinos expressed greater positivity (and felt more similar) toward Black Americans after the racial discrimination suffered by their own group was made salient (see also Bukowski et al., 2019). One likely explanation for this effect is that respondents experienced the commonalities with the other discriminated group, thus forming a superordinate category of individuals who are victims of racial discrimination which, in turn, fosters solidarity and more positive attitudes (Gaertner and Dovidio, 2000). In sum, current models suggest that sharing or not the dimension along which two minority groups are stigmatized is one of the key variables that may either enhance or reduce reciprocal negative attitudes (Craig and Richeson, 2016).

Critically, the aforementioned studies mainly focused on how intra-minority attitudes shift as a function of making salient or not the fact that one’s group is indeed victim of stigmatization. However, less is known about whether the overall levels of prejudiced attitudes toward other stigmatized minorities differ as compared to those hold by members of a majority group. A first relevant study aimed at filling this gap (Burson and Godfrey, 2018) has shown that racial, sexual, and gender minority groups in the United States tend to display more positive attitudes toward other marginalized minorities. There is thus preliminary evidence that intra-minority attitudes are somewhat more positive as compared to majority-minority attitudes. The analysis of this overall pattern may be of particular interest in the case of groups that are confronted with chronic experiences of strong stigmatization, such as people suffering from schizophrenia (Grover et al., 2017). Based on the findings reported by Burson and Godfrey (2018), it could be expected that individuals with a diagnosis of schizophrenia would show reduced level of negative attitudes toward other minorities as compared to the attitudes of non-clinical individuals belonging to the majority group.

### Perception of Social Groups in Schizophrenia

Social cognition in schizophrenia is often significantly altered (Penn et al., 2008; Green and Horan, 2010) and deficits can be found, for instance, in relation to emotion processing, attributional processes, and theory of mind (Green et al., 2012). In addition, there is an altered understanding of the rules that govern actual social interactions (Addington and Piskulic, 2011; Savla et al., 2013). However, several aspects of more declarative social knowledge appear to be largely preserved (Langdon et al., 2014; see also Ochsner, 2008) and this has important implications in relation to the perception that people with schizophrenia have toward social groups.

People suffering from schizophrenia are often the target of largely negative attitudes and behaviors (e.g., Grover et al., 2017; Mannarini et al., 2018) and they are also well aware of the stigma that is associated to their group membership (Rüsch et al., 2010). In addition, the knowledge that individuals with schizophrenia have about how they are perceived by other people appears to be one of the factors that contributes to exacerbate their impaired social functioning (Henry et al., 2010). In sum, there appears to be a good knowledge about how their own group is perceived by other individuals around them.

Recent evidence suggests that also the perception of people with schizophrenia toward other stigmatized groups is largely aligned to the culturally shared stereotypes associated to such groups. Castelli et al. (2017) have shown that there is a strong overall overlap in the stereotypical traits that individuals with a diagnosis of schizophrenia and non-clinical individuals attribute to Black immigrants. In other words, the most likely attributed traits by respondents in the clinical and control group were basically the same. Despite the consensus about what the typical traits of the target group were (e.g., aggressive and lazy), respondents in the clinical and control group differed in their personal beliefs about the extent to which these traits were actually prevalent among target group members (e.g., the percentage of Black immigrants who could be described as aggressive, lazy, and so on). Notably, participants with a diagnosis of schizophrenia were less likely than the control group to perceive Black immigrants as having negative stereotypical traits, thus suggesting a general more positive attitude. Consistent results were obtained when considering Gipsy people as a target group (Castelli et al., 2017).

In the present research we thus aimed to further explore whether individuals with a diagnosis of schizophrenia do actually consistently express less negative attitudes toward a stigmatized racial outgroup. More specifically, we attempted to assess attitudes in a relatively easier way that do not require complex abstract thinking as the one involved when asking to report the percentage of Black immigrants who could be described with various personality traits (see Castelli et al., 2017). For this reason, participants were presented with several pictures of White and Black targets and asked, on the basis of their gut reactions, to pick up the picture of the person who they believed possessed specific features to a greater extent (e.g., Who is the nicest? Who is the least trustworthy?). On the basis of existing findings from previous studies (Castelli et al., 2017; Burson and Godfrey, 2018), we predicted that individuals with a diagnosis of schizophrenia would display less negative attitudes.

## Study 1

### Methods

#### Participants

Eleven White outpatient clinical participants (three females, eight males) took part in the study. Diagnosis of schizophrenia was made by a board-certified attending research team of psychiatrists using the International Classification of Diseases (ICD-10). They were all under antipsychotic medication treatment. The age range was 31–62 years (*M* = 53.0, SD = 8.94). Eleven White non-clinical participants who perfectly matched the clinical sample in terms of gender, and with a similar age (range 31–65 years, *M* = 53.54, SD = 9.34, *t* = 0.14; *p* > 0.89) were also recruited. They reported neither personal nor family history of psychiatric/neurological illness.

#### Procedure

Both participants in the clinical and control group followed the same procedure. They were shown 18 pictures portraying an equal number of Black and White individuals and asked a series of questions. First, participants were asked to indicate the person who was judged as the most physically pleasant and the person who was judged as the most physically unpleasant. Next, participants were enquired about the target they considered as the nicest one. Two further questions required indicating the target person they considered as the most trustworthy and the one they perceived as the least trustworthy. One question concerned the target person who was perceived as the most likely to have committed a theft. Two additional questions required indicating the target person participants would have preferred to know and the one they would have not liked to know. Overall, there were thus four questions tapping positive dimensions and four questions tapping negative dimensions. Finally, two control questions assessed the perception of similarity and wealth: Participants had to indicate the target they perceived as more similar to them and the target they perceived as the wealthiest one.

##### Results and discussion

Preliminary chi-square analyses have been carried on the responses from each specific item aimed at assessing personal attitudes. Significant effects emerged in relation to judgments about the target who was considered as the nicest one, χ^2^(1) = 6.10, *p* < 0.05: Indeed, a White person was more likely selected by the control group as compared to the clinical group. Significant effects also emerged in relation to the most physically unpleasant target, χ^2^(1) = 3.66, *p* = 0.05, and about the target they would have not liked to know, χ^2^(1) = 6.60, *p* < 0.05. In both cases, a Black target was more likely selected by the control group. For all the other items no statistically significant effect was detectable.

In order to obtain a more reliable index, a global attitude score was computed. For each participant we calculated the number of times a response signaled a relative preference for Black targets (i.e., a positive feature was assigned to Black targets or a negative feature was assigned to White targets). This global score (α = 0.64)^1^ could range from 0 (extreme preference for White targets) to +8 (extreme preference for Black targets). A *t*-test indicated a significant difference between the responses of the two groups of respondents, *t*(20) = 2.26, *p* = 0.028, *d* = 1.01. Indeed, participants in the clinical group displayed an overall more positive attitude toward Black targets (*M* = 3.36, SD = 1.56) as compared to the non-clinical group (*M* = 1.72, SD = 1.67)^2^.

Finally, we analyzed the data about the two control questions (i.e., perception of similarity and wealth). All participants indicated a White target as the one most similar to them. No difference also emerged when asked to indicate the wealthiest target, χ^2^ (1) = 1.3, *p* > 0.25. Thus, it appears that participants in the clinical group have clear awareness about their belongingness to the specific group of White people and about the fact that Black individuals have objective lower wealth opportunities as compared to White individuals.

The overall pattern of findings indicated that intergroup attitudes were less negative in the clinical group. In Study 2 we explored what lay people believe about the attitudes toward Black people hold by individuals with a diagnosis of schizophrenia. Previous work has shown that majority group members tend to expect stigmatized minorities, as compared to majority groups, to be more tolerant toward other disadvantaged groups (Fernández et al., 2014). Accordingly, it could be predicted that similar expectations do also apply to the perception of people with schizophrenia. To this end, participants were presented with a questionnaire with a structure that largely resembled the assessment setting adopted in Study 1, and they were asked to separately predict the responses of a sample of individuals with a diagnosis of schizophrenia and the responses of a sample of non-clinical individuals with no history of mental disorders.

## Study 2

### Methods

#### Participants

One-hundred and eighty respondents, recruited through social networks, participated in the study (*M*_*age*_ = 29.41 years, SD = 12.91; Range 18–70 years; 79.9% females).

#### Procedure

Participants filled in an online questionnaire administered through Qualtrics after having provided an informed consent. They were explained that we had carried out a research comparing the responses of a sample of individuals with a diagnosis of schizophrenia and the responses of a sample of individuals with no history of mental disorders. They were later presented with the same 18 pictures used in Study 1 and asked to predict the responses of those two groups when asked to report the three individuals who were considered as (a) the most physically pleasant, (b) the most physically unpleasant, (c) the most amiable, (d) the most trustworthy, (e) the most untrustworthy, (f) the most desirable to know, (g) the most likely to have committed a theft, and (h) the least desirable to know. At the end, participants were also asked to report their gender, age, whether they were involved or had been involved in any academic curriculum and, in such case, in what specific field.

##### Results and discussion

Because in some cases respondents did not report the exact required number of pictures (i.e., three for each question), we first calculated, for each question, the proportion of times in which a Black person was indicated. Some participants did not provide an answer to all the questions and therefore the degrees of freedom vary accordingly. As a general strategy for comparing the predicted responses of individuals with schizophrenia versus non-clinical individuals, when the responses were normally distributed parametric analyses were carried out, otherwise non-parametric tests were performed.

As for the prediction of judgments about the most physically pleasant targets, a Wilcoxon test showed that individuals with schizophrenia (versus non-clinical individuals) were expected to more likely indicate Black targets, *Z* = −3.60, *r* = −0.27 (see Table 1). No significant effects were found for judgments about the least physically pleasant targets and the perceived nicest targets.

**TABLE 1:** Predictions in Study 2.

**Type of judgment**	**Individuals with schizophrenia**	**Healthy individuals**
Physical pleasantness	0.1608 (0.248) A	0.1039 (0.194) B
Physical unpleasantness	0.5414 (0.310) B	0.5645 (0.254) A
Amiability	0.3439 (0.272) A	0.3170 (0.269) B
Trustworthiness	0.2590 (0.290) A	0.2016 (0.259) B
Untrustworthiness	0.6019 (0.312) B	0.6369 (0.295) A
Involved in a thief	0.6682 (0.320) B	0.6828 (0.272) A
Desire to know	0.2517 (0.307) A	0.1748 (0.245) B
Desire not to know	0.5865 (0.323) B	0.6115 (0.286) A

*Proportion of responses indicating a Black person, as a function of the group participants had to predict the responses. Standard deviations are reported in parentheses. Letters “A” and “B” indicate how the proportion values have been combined in order to compute the overall attitude score. All the four subscales related to the predicted attributions of clinical and non-clinical people for either positive or negative features displayed satisfactory reliability as calculated with SPPS Categories procedure CATPCA (αs > 0.72; Meulman et al., 2004).*

The predictions about trustworthiness were analyzed through a 2 (group: individuals with schizophrenia versus non-clinical) × 2 (dimension: trustworthiness versus untrustworthiness) analysis of variance. A strong main effect of the dimension emerged, *F*(1,147) = 144.33, *p* < 0.001, η*_*p*_*^2^ = 0.495. Indeed, participants expected that a Black person would be more likely indicated in the case of the negative trait as compared to the positive trait (*Ms* = 0.62 and 0.23, SE*s* = 0.021 and 0.019, respectively). The main effect of the group was not significant. The key interaction effect was significant, *F*(1,147) = 5.57, *p* = 0.020, η*_*p*_*^2^ = 0.036. Participants tended to expect that when evaluating trustworthiness, a Black person would be more likely picked up by individuals with schizophrenia, as compared to non-clinical individuals, *p* = 0.06, whereas the opposite, although non-significant, pattern emerged for the evaluation of untrustworthiness (see Table 1).

As for the prediction of judgments about the persons who could have being involved in a thief, a *t*-test did not show any significant effect.

In relation to the predictions about the willingness to know the Black targets, a Wilcoxon test showed that individuals with schizophrenia (versus non-clinical individuals) were expected to more likely indicate Black targets, *Z* = −2.89, *r* = −0.24. The effect about the desire to not know Black target was not statistically significant.

Because the overall pattern of findings was consistent across the various measures (see Table 1) although the effects did not always reach the conventional level of significance, we finally computed a single score about the predicted attitude toward Black targets hold by individuals with a diagnosis of schizophrenia as compared to that hold by non-clinical individuals (i.e., values marked with an “A” minus values marked with a “B” in Table 1)^3^. In this way, positive scores indicate that people with schizophrenia are expected to hold more positive attitudes than non-clinical people. The observed value was significantly higher than zero, *t*(135) = 3.36, *p* = 0.001, *d* = 0.288, *M* = 0.35, and *SD* = 1.23, demonstrating that participants expected that individuals with a diagnosis of schizophrenia would display an overall more positive attitude toward Black people. Age was not correlated with this score (*p* = 0.85). The gender of the participant did not affect responses, as well as the fact that the respondent was involved (or had been involved) in an academic curriculum (*ts* < 1, *ps* > *0.50*). In an exploratory way, we also assessed whether being involved in studying psychology (*N* = 42) or other disciplines (*N* = 94) was a relevant factor, assuming that psychology students might have better knowledge about schizophrenia. No difference emerged (*t* < 1, *p* > *0.73*).

Overall, lay persons believe that individuals with a diagnosis of schizophrenia tend to have more positive attitudes toward Black people as compared to non-clinical individuals who do not suffer from mental disorders. Thus, it appears that lay perceivers provide relatively accurate predictions about the relative attitudes hold by the two groups.

## General Discussion

Research has only recently started to explore how people suffering from mental illnesses perceive the various social groups around them. The available evidence suggests that, despite severe impairments in several aspects of social cognition, there is a relatively intact declarative knowledge about the stereotypical features associated to stigmatized social groups. For instance, children affected by autism report a good knowledge about both race and gender stereotypes, and their responses do not differ from those displayed by non-clinical children (Hirschfeld et al., 2007; Da Fonseca et al., 2010; see also Birmingham et al., 2015). In a similar vein, the responses of adult people with a diagnosis of schizophrenia about the culturally shared stereotypes associated to Black and Gipsy individuals do not qualitatively differ from the responses of control non-clinical adults (Castelli et al., 2017; see also Champagne-Lavau and Charest, 2015). Having similar representations concerning the stereotypically associated traits, however, does not necessarily imply that the overall valence of the attitudes is also similar. In the present work we further focused on the attitudes toward Black individuals hold by people with schizophrenia. As discussed in the introduction section, making discrimination against the in-group salient may significantly affect intra-minority perceptions (Craig and Richeson, 2012, 2014; Bukowski et al., 2019). The present findings corroborate the conclusion that even without any reminder about discrimination against the in-group, attitudes toward another discriminated group may be less negative as compared to the attitudes hold by the majority group (Castelli et al., 2017; Burson and Godfrey, 2018). Indeed, individuals with schizophrenia reported less negative attitudes toward Black people as compared to the control group, even though the criterion defining group membership (i.e., race) was different from the one characterizing their status as a stigmatized group (i.e., presence of a mental disorder). Interestingly, the results from Study 2 largely support the idea that lay people also have naïve intuitions about reduced levels of prejudiced attitudes among individuals with a diagnosis of schizophrenia. This latter finding is consistent with the results reported by Fernández et al. (2014) who showed that stigmatized group members are associated to demanding moral standards that include more tolerant attitudes toward other minorities. It can be tentatively suggested that also people suffering from schizophrenia are confronted with expectations from people around them to behave in accordance to stricter moral standards and, therefore, to be more tolerant toward other disadvantaged groups. As an alternative explanation, it might simply be that lay people consider individuals with a diagnosis of schizophrenia as less able to integrate information about categorical membership in their social judgments, thus reducing the differentiation in the responses toward different social groups.

Findings from Study 1 are consistent with the idea that people suffering from schizophrenia do not necessarily rely on the derogation of other stigmatized groups to bolster self-esteem (Hogg and Abrams, 1990). In contrast, findings appear to be more in line with a general more positive perception of other groups that are targets of social discrimination (see Burson and Godfrey, 2018). One possibility is that individuals suffering from a severe mental disorder, such as schizophrenia, that is immediately evident in any social interaction, are more subject to chronic experiences of stigmatization (e.g., Grover et al., 2017) and this makes them more sensitive to the negative experiences that members of other stigmatized groups may also face. In other words, the shared experience of being consistently stigmatized might have enhanced the perception of commonalities, fostering more positive attitudes (Gaertner and Dovidio, 2000; Burson and Godfrey, 2020). The overarching criterion for creating a common identity may thus be the shared status as stigmatized groups, independently from the specific features that lead to such stigmatization (Cortland et al., 2017; see also Chaney et al., 2018).

### Limitations and Directions for Future Research

Future research will have to assess the strength of the obtained findings by testing larger and representative samples. One major shortcoming of Study 1 is indeed related to the limited sample size which makes the study underpowered to draw definite conclusions, although findings were consistent with previous research (see Castelli et al., 2017). The issue of generalizability also represents a key limitation. On the one hand, findings should be replicated with novel and pretested face stimuli. Although the major interest here was about the responses of different groups toward the same set of pictures, the use of pretested and validated sets will allow to achieve a better experimental control. On the other hand, studies focusing on intra-minority attitudes in schizophrenia are, so far, based on the perception of out-groups for which their stigmatized status is ascribed (e.g., racial/ethnic membership). It will be important to disentangle whether people with schizophrenia do actually hold a pervasive more positive perception of all stigmatized out-groups, or this is confined to conditions of ascribed group memberships, namely when the status is assigned at birth or assumed involuntarily later in life. To this end, future studies might include other target groups that are subject to derogatory attitudes because of their voluntarily performed behaviors. First, this will enable to assess whether individuals who suffer from schizophrenia have more positive social attitudes in general, or they are more benevolent only toward groups facing discrimination for reasons independent from their actual will. In addition, this will allow to more thoroughly explore the social reasoning that characterize people with a diagnosis of schizophrenia in relation to the various motivations that may lead to the stigmatization of different social groups, thus allowing to more directly focus on the processes underlying the effects observed in the present research.

Future research will also have to specifically investigate the perception toward individuals suffering from other types of mental disorders (e.g., anorexia nervosa). Current models and empirical data (Craig and Richeson, 2016; Burson and Godfrey, 2018) would lead to predict that people with schizophrenia, as compared to non-clinical individuals, might hold even more positive attitudes toward such groups because of the commonalities with them that should further facilitate the creation of an overarching common social identity.

As discussed in the introduction section, previous research addressing intra-minority attitudes were mainly based on the comparison between the responses of minority group members who were either reminded of their stigmatized status or not. An empirical question is thus related to the potential effects among people suffering from mental illness of priming past personal experiences of discrimination: this might further increase positive (or less negative) attitudes toward other stigmatized groups or, in sharp contrast, it might prompt a tendency to contextually derogate the out-groups in order to bolster one’s self esteem that has just been threatened (Cadinu and Reggiori, 2002).

Finally, although previous research in this area indicates that the formation of an overarching shared identity of stigma represents a key underlying process, its involvement in the case of the specific group of people who are stigmatized because of their mental health condition has to be tested yet.

## Conclusion

Research about the attitudes toward stigmatized social groups hold by people affected by schizophrenia is so far scarce, but the available evidence consistently indicates that such attitudes tend to be less negative as compared to those hold by non-clinical individuals. People with schizophrenia, who are victims of chronic experiences of stigmatization, thus appear to be more benevolent toward other social groups that also repeatedly faces social discrimination, and this pattern is also reflected in the naïve intuitions and expectations of lay people.

## Data Availability Statement

The raw data supporting the conclusions of this article will be made available by the authors, without undue reservation.

## Ethics Statement

The studies involving human participants were reviewed and approved by Ethics Committee for Psychology–University of Padua. The patients/participants provided their written informed consent to participate in this study.

## Author Contributions

LCas, LCar, CU, AV, and MP conceived the studies. LCas performed the analyses. LCas, LCar, CU, and AV wrote the manuscript. All authors contributed to the article and approved the submitted version.

## Conflict of Interest

The authors declare that the research was conducted in the absence of any commercial or financial relationships that could be construed as a potential conflict of interest.

## Footnotes

1 Reliability was calculated with SPPS Categories procedure CATPCA (Meulman et al., 2004).

2 A non-parametric analysis on the global attitude score yielded a similar finding (*U* = 29, *p* = 0.04). A sensitivity analysis with G-Power indicated that the study had 80% power to detect a large effect size (i.e., *d* = 1.2).

3 A sensitivity analysis with G-Power indicated that the study had 80% power to detect a relatively small effect size (i.e., *d* = 0.24).
